# Supplementary material for: Comparison of Characteristics and Outcomes of Multisystem Inflammatory Syndrome, Kawasaki Disease and Toxic Shock Syndrome in Children
Source: Medicina (Kaunas). 2023 Mar 21;59(3):626. doi: 10.3390/medicina59030626 (PMC10056689; doi:10.3390/medicina59030626)
Supplement: Supplementary file 1 [file medicina-59-00626-s001.zip › Supplementary Material 1..pdf]

Supplementary Material 1: Distribution of clinical characteristics of patients with KD, MIS-C and TSS

Clinical characteristics	KD	MIS-C	TSS	Total	*P*-value	KD vs. MIS-C, *P*-value	MIS-C vs. TSS, *P*-value	KD vs. TSS, *P*-value
Dermatological/mucocutaneous involvement								
Conjunctivitis, n (%)	35 (89.7)	24 (82.8)	7 (53.8)	66 (81.5)	**0.02**	0.48	0.07	**0.01**
Changes in lips and oral cavity, n (%)	36 (97.3)	28 (96.6)	12 (92.3)	76 (96.2)	0.57			
Polymorphous exanthema, n (%)	37 (94.9)	26 (89.7)	13 (100.0)	76 (93.8)	0.57			
Changes in extremities, n (%)	25 (73.5)	16 (55.2)	10 (76.9)	51 (67.1)	0.22			
Cervical lymphadenopathy, n (%)	25 (75.8)	12 (41.4)	2 (15.4)	39 (52.0)	**<0.001**	**0.006**	0.16	**<0.001**
Gastrointestinal involvement								
Abdominal pain, n (%)	9 (23.1)	23 (79.3)	5 (38.5)	37 (45.7)	**<0.001**	**<0.001**	**0.02**	0.30
Diarrhoea, n (%)	10 (25.6)	19 (65.5)	4 (30.8)	33 (40.7)	**0.003**	**0.001**	**0.04**	0.73
Vomitting, n (%)	13 (33.3)	16 (55.2)	9 (69.2)	38 (46.9)	**0.04**	**0.07**	0.39	**0.02**
Respiratory symptoms								
Cough, n (%)	22 (56.4)	13 (44.8)	6 (46.2)	41 (50.6)	0.60			
Shortness of breath, n (%)	8 (20.5)	14 (48.3)	6 (46.2)	28 (34.6)	**0.04**	**0.02**	0.90	0.09
Cardiovascular symptoms								
Shock, n (%)	7 (17.9)	18 (62.1)	13 (100.0)	38 (46.9)	**<0.001**	**<0.001**	**0.01**	**<0.001**
Hypotension, n (%)	2 (5.1)	16 (55.2)	10 (76.9)	28 (34.6)	**<0.001**	**<0.001**	0.30	**<0.001**
Musculoskeletal symptoms								
Arthralgias, n (%)	9 (23.1)	1 (3.4)	1 (7.7)	11 (13.6)	**<0.001**	**<0.001**	0.47	**0.007**
Synovitis, n (%)	28 (71.8)	0	1 (7.7)	29 (35.8)		0.99	-	**<0.001**
Myalgias, n (%)	0	5 (17.2)	3 (23.1)	8 (9.9)		-	0.52	-
Neurologic symptoms								
Headache, n (%)	7 (17.9)	15 (51.7)	4 (30.8)	26 (32.1)	**0.01**	**0.003**	0.21	0.44
Neck stiffness, n (%)	1 (2.6)	5 (17.2)	2 (15.4)	8 (9.9)	0.08			
Photophobia, n (%)	3 (7.7)	2 (6.9)	2 (15.4)	7 (8.6)	0.66			
Meningism, n (%)	1 (2.6)	2 (6.9)	2 (15.4)	5 (6.2)	0.23			

Hyperesthesia, n (%)	3 (7.7)	8 (27.6)	1 (7.7)	12 (14.8)	0.07
