# Supplementary material for: Comparison of Characteristics and Outcomes of Multisystem Inflammatory Syndrome, Kawasaki Disease and Toxic Shock Syndrome in Children
Source: Medicina (Kaunas). 2023 Mar 21;59(3):626. doi: 10.3390/medicina59030626 (PMC10056689; doi:10.3390/medicina59030626)
Supplement: Supplementary file 1 [file medicina-59-00626-s001.zip › Supplementary Material 2..pdf]

Supplementary Material 2: Characteristics of laboratory analyses and other investigations in patients with MIS-C, KD and TSS

Laboratory test	KD n=39	MIS-C n=29	TSS n=13	Total	*P*-value	KD vs. MIS-C, *P*-value	MIS-C vs. TSS, *P*-value	KD vs. TSS, *P*-value
Total white blood cell count (x10^3/µl), median (IQR)	13.0 (10.6-17.6)	7.3 (5.1-10.0)	16.7 (11.1-21.1)	11.2 (7.5-16.5)	**<0.001**	**<0.001**	**<0.001**	0.26
Absolute neutrophil count initially (x10^3/µl), median (IQR)	9.1 (6.9-11.9)	5.6 (4.0-8.4)	15.1 (9.5-19.5)	8.5 (5.7-12.7)	**<0.001**	**0.001**	**<0.001**	**0.007**
Relative neutrophil count (%), median (IQR)	67.7 (60.0-77.5)	83.8 (76.1-87.7)	92.1 (83.9-94.7)	77.6 (64.8-87.7)	**<0.001**			
Absolute lymphocyte count initially (x10^3/µl), median (IQR)	1.9 (1.3-4.0)	0.6 (0.5-0.8)	0.5 (0.2-0.7)	1.0 (0.5-2.1)	**<0.001**	**<0.001**	**0.02**	**<0.001**
Platelet count initially (x10^3/µl), median (IQR)	328 (226-463)	130.0 (104.0-178.5)	209.0 (169.0-251.5)	221.0 (132.5-329.5)	**<0.001**	**<0.001**	**0.03**	**0.001**
Anemia, n (%)	11 (28.2)	15 (51.7)	3 (23.1)	29 (35.8)	0.08			
Sodium level initially (mmol/l), mean (SD)	137.4 (3.3)	132.9 (4.6)	131.0 (4.5)	134.6 (4.8)	**<0.001**	**<0.001**	0.23	**<0.001**
Albumin level (g/l), mean (SD)	31.9 (7.4)	27.9 (5.8)	24.9 (4.2)	29.9 (7.0)	**0.03**	**0.03**	0.32	0.07
ESR (mm/h), mean (SD)	40.8 (29.7)	54.2 (36)	11.8 (35.8)	40.9 (35.6)	**0.001**	0.1	**0.001**	**0.006**
CRP (mg/l), median (IQR)	74.0 (47.0-124.0)	167.9 (106.0-271.0)	160 (115.2-234.5)	117.1 (64.1-193.4)	**<0.001**	**<0.001**	0.79	**0.001**
Interleukin-6 (pg/ml), median (IQR)	93.1 (38-208.3)	176.0 (94.8-383.5)	134.0 (46.9-406.3)	139.0 (59.5-297.5)	0.1			

Ferritin (ng/ml), median (IQR)	207.5 (146.1-360.4)	543.3 (288.0-733.3)	149.0 (110.9-196.1)	299.0 (168.7-545.3)	**<0.001**	**<0.001**	0.13	0.07
Lactate dehydrogenase (U/l), median (IQR)	258.0 (246.5-299.8)	273.5 (335.0-328.8)	337.0 (229.0-351.5)	273.0 (237.0-329.0)	0.76			
D-dimers (mg/l FEU), median (IQR)	2.7 (0.9-4.8)	3.3 (2.5-6.8)	3.7 (2.3-5.7)	3.1 (1.9-4.9)	0.08			
Fibrinogen (g/l), median (IQR)	4.0 (3.1-5.2)	5.2 (4.2-6.3)	4.7 (3.1-5.8)	4.4 (3.7-5.7)	**0.04**	**0.02**	**<0.001**	0.66
High-sensitivity Troponin I level (ng/ml), median (IQR)	0.01 (0.01-3.1)	42.3 (15.7-120.4)	6.7 (3.3-12.7)	8.6 (0.02-36.6)	**<0.001**	<0.001	**0.002**	**0.03**
NT-proBNP (pg/ml), median (IQR)	1526 (442-4107)	5318.4 (1950.1-13312.6)	1733.8 (245.0-1733.8)	3189.2 (1423.8-8186.8)	**0.008**	0.005	0.08	0.57
Increased liver enzymes, n (%)	19 (50)	14 (48.3)	10 (76.9)	43 (53.1)	0.17			
Acute kidney injury, n (%)	3 (7.7)	4 (13.8)	6 (46.2)	13 (16.0)	**0.01**	0.45	**0.046**	**0.005**
